# Fellowship of the Australian College of Rural & Remote Medicine (FACRRM) Assessment: a review of the first 12 years

**DOI:** 10.15694/mep.2020.000100.1

**Published:** 2020-05-18

**Authors:** Tarun Sen Gupta, David Campbell, Alan Bruce Chater, David Rosenthal, Lynn Saul, Karen Connaughton, Marita Cowie

**Affiliations:** 1 James Cook University and Australian College of Rural and Remote Medicine; 2 Australian College of Rural and Remote Medicine

**Keywords:** rural medicine, assessment, certification, licensing exam, tele-assessment

## Abstract

This article was migrated. The article was marked as recommended.

This paper provides an overview of the first 12 years of the formal assessment program of the Australian College of Rural and Remote Medicine (ACRRM). The ACRRM Fellowship represents the world’s first and only Fellowship exam in Rural Medicine.

The ACRRM assessment program is mapped to its Rural Generalist curriculum, based on the principles of programmatic assessment. ACRRM offers candidates the opportunity to participate in assessment in or close to their home location. The ACRRM Rural Generalist Curriculum defines the scope and standards for independent general practice anywhere in Australia, with a focus on rural and remote settings.

The program was initially developed in 2006 and has evolved during delivery from 2008 onwards, utilising the following modalities:

• Multi Source Feedback (MSF)
• Multiple Choice Questions (MCQ)
• Mini Clinical Evaluation Exercise (Mini-CEX)
• Case Based Discussion (CBD)
• Procedural Skills Logbook
• Structured Assessment using Multiple Patient Scenarios (StAMPS)

StAMPS is a unique examination, blending the formats of an Objective Structured Clinical Examination and a traditional viva vocè.

The program has an emphasis on formative assessment. Over the past 12 years there has been considerable work in developing resources for candidates, governance structures and quality assurance processes.

ACRRM’s Fellowship requirements represent a customised bespoke assessment tailored to ACRRM’s curriculum and the Australian rural and remote context. ACRRM’s assessment program has grown substantially with 649 Fellowships being awarded from 2008 - 2019, with considerable experience gained in rural and remote assessment. It now represents a mature firmly-established process as a vocational endpoint in Rural and Remote Medicine.

ACRRM has continued to offer its ‘tele-assessment’ program throughout the COVID-19 pandemic, with candidates and examiners participating in assessment by use of distance technology while remaining in or near their home community. This model may provide some insights for other medical Colleges and educational institutions facing challenges in the current environment.

## Context

This paper describes the implementation of the first 12 years of the formal assessment program of the Australian College of Rural and Remote Medicine (ACRRM). The ACRRM Fellowship (FACRRM) represents the world’s first and only Fellowship exam in Rural Medicine. It represents a vocational endpoint accredited by the Australian Medical Council that leads to Specialist Registration as a General Practitioner, signifying certification as a specialist in rural and remote medicine. Training may be completed through two commonwealth funded pathways, the Australian General Practice Training Program and the Remote Vocational Training Scheme, or a self-funded Independent Pathway delivered directly by the College.

ACRRM was incorporated in March 1997 with Fellowship criteria advertised to foundation members in 1998 and a formal assessment program leading to Fellowship established in 2008 ( Australian College of Rural and Remote Medicine, 2020a). ACRRM’s principles of assessment remain consistent with those developed at this time ( Smith *et al.*, 2007). This paper traces the approaches to assessment of the Rural Generalist Curriculum and implementation over the past 12 years

The ACRRM Vocational Training Program comprises four years full-time training, consisting of three years Core Generalist Training (previously known as the Primary Curriculum, which included 12 months Core Clinical Training and 24 months Primary Rural and Remote Training) in accredited hospitals and a range of rural community primary care and hospital facilities, and one year Advanced Specialised Training (AST) in one of eleven disciplines ( Australian College of Rural and Remote Medicine, 2020b).

The ACRRM Rural Generalist Curriculum defines the scope and standards for independent general practice anywhere in Australia, with a particular focus on rural and remote settings. It sets out the outcomes expected at ACRRM Fellowship level ( Australian College of Rural and Remote Medicine, 2020c). The eleven AST disciplines each have their own curriculum and assessment ( Australian College of Rural and Remote Medicine, 2020d). This paper focuses on assessment of the Core Generalist curriculum.

The College developed its assessment program based on two key principles:

• candidates have the opportunity to participate in assessment within the locality where they live and work, preventing depopulating rural and remote Australia of medical workforce (candidates and assessors) during assessment periods
• the content of the assessment is developed by clinically active rural and remote medical practitioners

The ACRRM assessment program was mapped to the curriculum, based on the principles of programmatic assessment, comprised of different assessment types covering all levels of Miller’s Pyramid ( Smith *et al*., 2007) and offered over the four years of training. The assessment standard for modalities requiring a pass grade was defined at that of a ‘general practitioner practising safely and independently in a rural or remote community’( Australian College of Rural and Remote Medicine, 2019, p6). The assessment program was designed to be academically rigorous, flexible, valid, reliable, fair, and with a positive educational impact ( Australian College of Rural and Remote Medicine, 2020c).

## A Fellowship in Rural and Remote Medicine: History and Evolution of the Program

The ACRRM Fellowship assessment program was developed by a consortium of rural doctors, academics and policy experts in 2006 and first implemented in 2008. The original assessment program consisted of five modalities ( Smith *et al*., 2007) as set out in Table 1:

• Multi Source Feedback (MSF)
• Mini Clinical Evaluation Exercise (Mini-CEX)
• Multiple Choice Questions (MCQ)
• Structured Assessment using Multiple Patient Scenarios (StAMPS)
• Procedural Skills Logbook

**Table 1.** Core Generalist Curriculum Summative Assessment Requirements: 2008 and 2020 comparison

2008 Requirements	2020 Requirements
Multiple choice questions (MCQ)- pass grade required	Multiple choice questions (MCQ)- pass grade required
Multi-source feedback (MSF) - pass grade required	Multi-source feedback (MSF) - satisfactory completion required
	Case Based Discussion (CBD) - pass grade required
Structured Assessment using Multiple Patient Scenarios (StAMPS) - pass grade required	Structured Assessment using Multiple Patient Scenarios (StAMPS) - pass grade required
Procedural Skills Logbook - satisfactory completion required	Procedural Skills Logbook - satisfactory completion required
Mini clinical evaluation exercise (Mini-CEX) - pass grade required	Mini clinical evaluation exercise (Mini-CEX) - completion required

The AST curricula and assessments were introduced in 2010-2011 and include formative assessments and either mini-CEXs or a project as the summative component.

The assessment program continues to be based on the guiding principles and assessment philosophy outlined above but with some changes over time.

Candidate feedback has led to increased delivery options. Initially MCQ was only offered in remote sites arranged by the candidate. Candidates now have the option to sit at a venue close to home or at a number of central assessment centres. Similarly, StAMPS was initially held only by videoconference; now candidates have the option of videoconference or face-to-face delivery.

Initially the MSF was graded (pass-fail). This was discontinued in 2012 due to challenges in determining the appropriate standard. Now successful completion and discussion with a medical educator is required.

Mini-CEX remains a central part of the assessment program but the focus has moved from a summative assessment conducted by external ACRRM assessors, to a formative assessment conducted by local supervisors and medical educators. This change resulted from challenges in recruiting external examiners and arranging travel, particularly for remote areas. Case Based Discussion (CBD) conducted via teleconference replaced summative mini-CEX in 2016.

The Core Generalist Curriculum assessment program currently (2020) has six summative components, summarised in Table 1 ( Australian College of Rural and Remote Medicine, 2020b). Formative assessment is integral to the program and includes regular supervisor feedback and reports, multiple observations of practice (eg, mini-CEX), reflection on practice (eg through MSF) and mock examinations and practice questions.

Candidates may undertake the assessments at any time during training providing they meet the minimum eligibility, which is one year of training for MSF and MCQ and two years for CBD and StAMPS. The recommended order is MSF, MCQ, CBD then StAMPS. Candidates are required to prospectively plan the timing of assessments and be guided by medical educators and supervisors to ascertain readiness ( Australian College of Rural and Remote Medicine, 2019). From 2020, candidates need to meet some additional requirements (pass in the MCQ assessment, completion of Mini-CEX formative assessment and/or MSF and completion of at least one formal StAMPS preparation activity) in order to sit the StAMPS examination. Further details on each modality follows.

## Multi Source Feedback (MSF)

Multi-Source Feedback (MSF) is a well-recognised, valid, and reliable method of assessing interpersonal and professional behaviour, development, and clinical skills ( Smith *et al.*, 2007). MSF provides candidates with feedback from patients and colleagues. Satisfactory completion of MSF requires evidence of an MSF report covering the two components, a reflective exercise, discussion with a Medical Educator, and remediation if required.

## Multiple Choice Questions (MCQ)

The MCQ examines knowledge recall, reasoning and applied clinical knowledge. The exam since inception has been conducted via the internet through a secure website using customised software (Question Mark ^TM^) which locks down the browser as a security measure. ACRRM offers the option to undertake this examination twice yearly within or close to the candidate’s own community or at a central examination centre. All candidates undertake the MCQ at the same time, regardless of their location.

The examination is conducted over three hours (180 minutes) and consists of 125 multiple choice questions delivered in a randomised manner for each candidate. Questions usually consist of a clinical case presentation, a brief targeted lead-in question and four options from which candidates are required to choose the single best option. The stem of the clinical case may include text and images.

**Figure 1.**
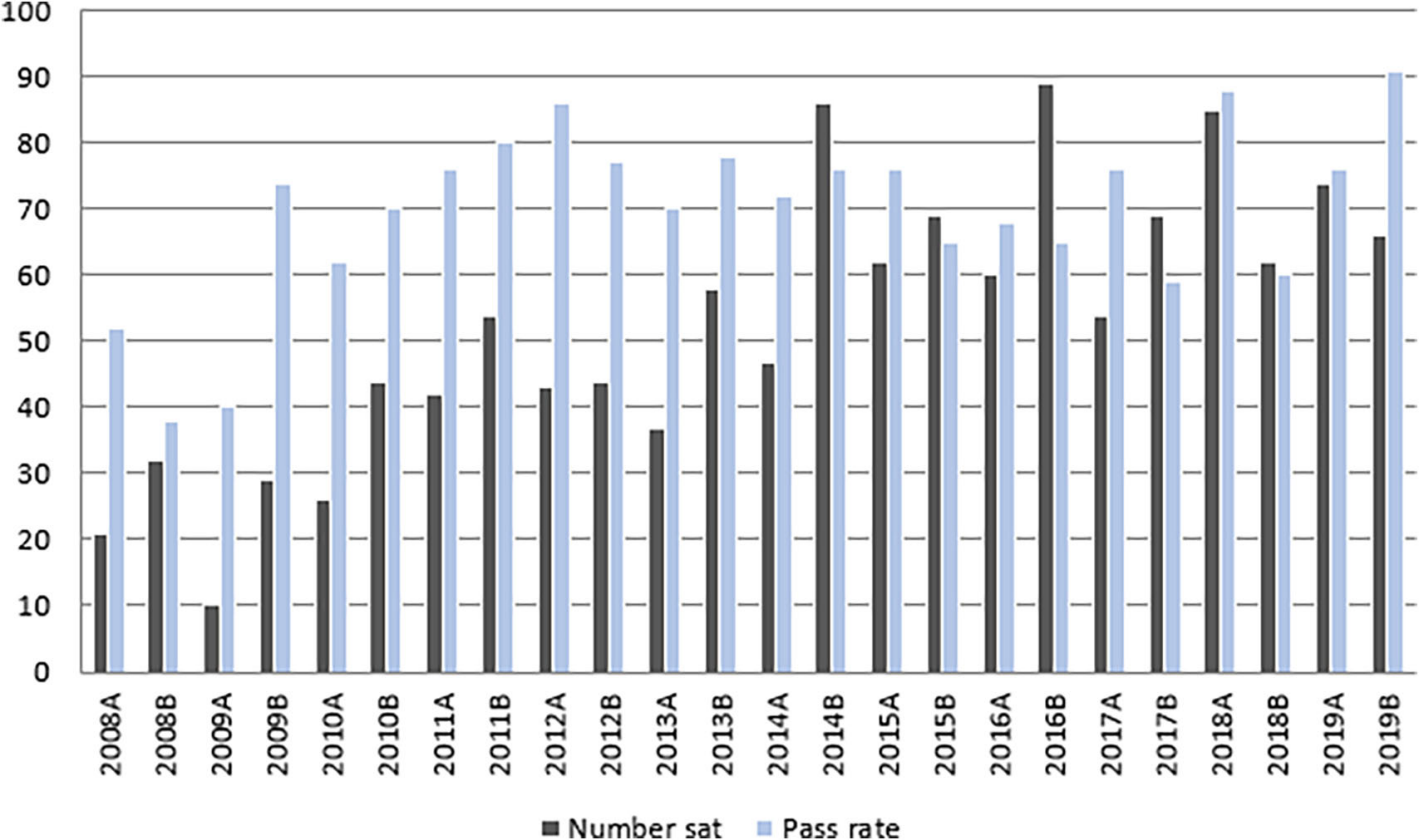
MCQ pass rate and participation 2008 - 2019

Figure 1 presents data on MCQ candidate numbers and pass rates from 2008-2019. 1061 candidates undertook the MCQ over this period with an overall pass rate of 68%. Sample MCQs are in Table 2.

**Table 2.** Sample Multiple Choice Questions (correct answers are asterisked)

Question 1
Margaret is a 50-year-old senior administrator who presents with R sided earache of one month’s duration. The pain radiates into the temple area and down the side of her face. The pain is worst when she wakes in the morning and after eating. On examination the ear is clear, there is lateral deviation of the jaw on opening and all other observations are normal. The **MOST** likely diagnosis is: a) Temporal arteritis b) Trigeminal neuralgia c) Temporomandibular joint dysfunction * d) Eustachian Tube Dysfunction **Question 2** You are managing a distressed five-year-old child who has been diagnosed with pneumonia in the emergency department of a small rural hospital. You wish to insert an IV cannula to administer antibiotics and collect blood. You decide to apply a local anaesthetic patch (2.5% prilocaine and 2.5% lignocaine, “EMLA”) How long will you need to wait, after the application of the patch to an appropriate site, before effective anaesthesia will allow pain free IV cannulation? a) 40 minutes b) 60 minutes * c) 90 minutes d) 120 minutes

## Mini Clinical Evaluation Exercise (Mini-CEX)

The Mini Clinical Evaluation Exercise (Mini-CEX) is a work-based assessment used to evaluate a candidate’s clinical performance in real-life clinical settings ( Smith *et al.*, 2007). Nine formative mini-CEXs are required. A mini-CEX can be conducted within the context of the candidate’s medical educator visit or at other convenient times.

The nine mini-CEX consultations must include a variety of content including:

• A minimum of 5 physical examinations, each from a different organ system,
• A detailed history from at least one new patient or detailed updating of patient database information on a returning patient (of at least medium complexity),
• A reasonable range of types of consultations, age groups and both genders.

**Figure 2.**
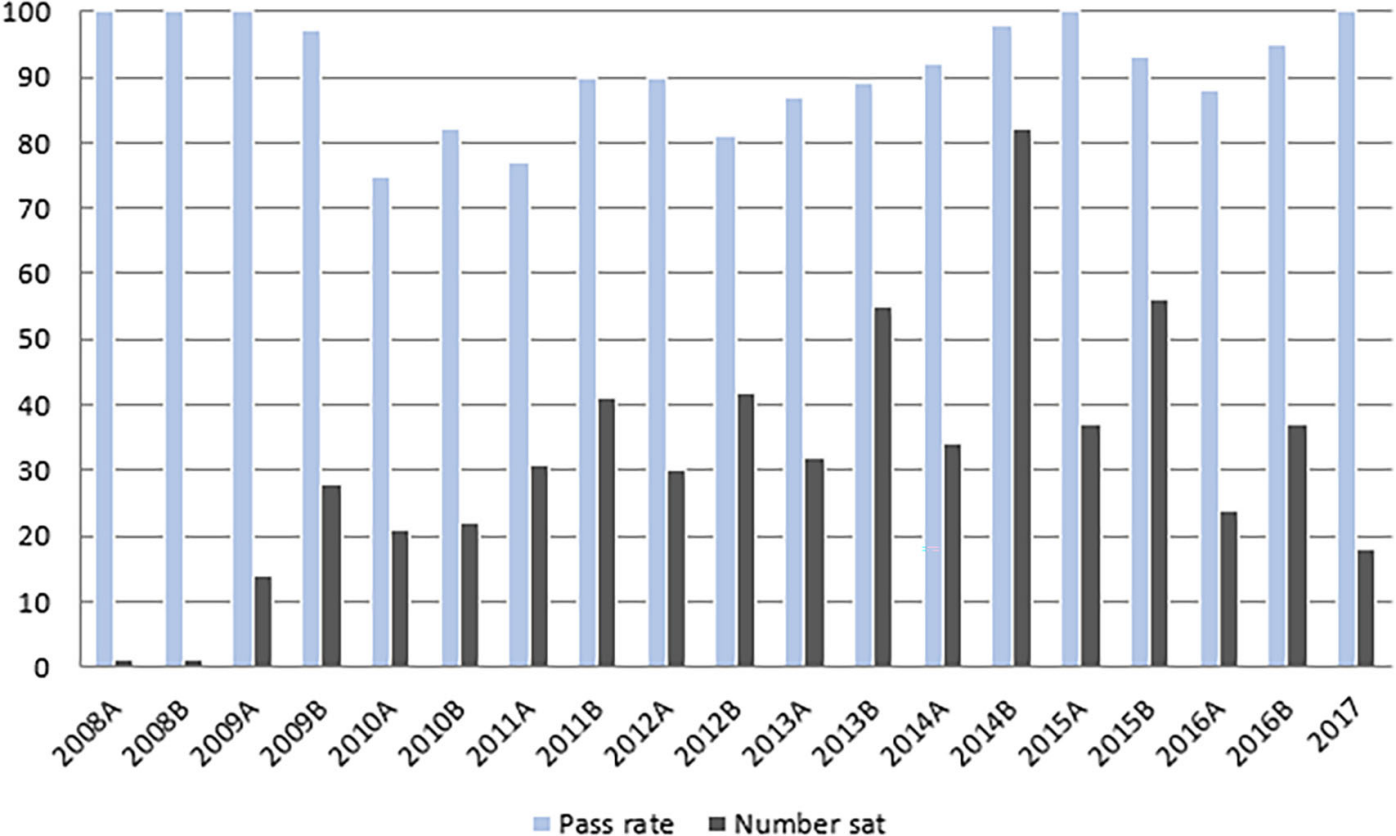
Mini-CEX pass rate and participation 2008 - 2017

The summative mini-CEXs (now discontinued for the logistical reasons mentioned above) had specific requirements for the qualifications and number of assessors. Figure 2 summarises summative mini-CEX numbers and pass rates from 2008-2017. 606 candidates undertook the mini-CEX over this period with an overall pass rate of 91%.

## Case Based Discussion (CBD)

Case Based Discussion, introduced in 2016, is an assessment of clinical reasoning and application of knowledge in a clinical context. The candidate needs to demonstrate evidence of their clinical knowledge and how they apply that knowledge by appropriately assessing patients, formulating differential diagnoses, ordering relevant investigations and developing appropriate management and follow up plans ( Brown, Holsgrove. and Teeluckdharry, 2011).

Candidates submit 12 de-identified cases, from which six are chosen. Cases must cover at least six different Core Generalist Curriculum clinical statements and must include one mental health consultation.

Cases should be obtained from a generalist service, Emergency or RFDS setting rather than a specialist service. Cases should be at least medium level of complexity in order to enable the candidate to demonstrate skills in clinical knowledge and reasoning.

The Case Based Discussion uses a format of 3 x 1-hour case discussions, usually by telephone, with the candidate in their home location and an assessor located anywhere in Australia. Different assessors are used for each session, with 2 cases selected for each session. An invigilator must be present to confirm the candidate’s identity and ensure appropriate security.

Five categories are graded for each case in order to determine the overall rating:

• Communication skills
• History taking
• Physical assessment
• Clinical Management in the rural/remote context
• Professionalism

Candidate must achieve a global CBD rating of ‘At expected standard for FACRRM’ in a minimum of five of the six CBD cases to be awarded a ‘pass grade’.

**Figure 3.**
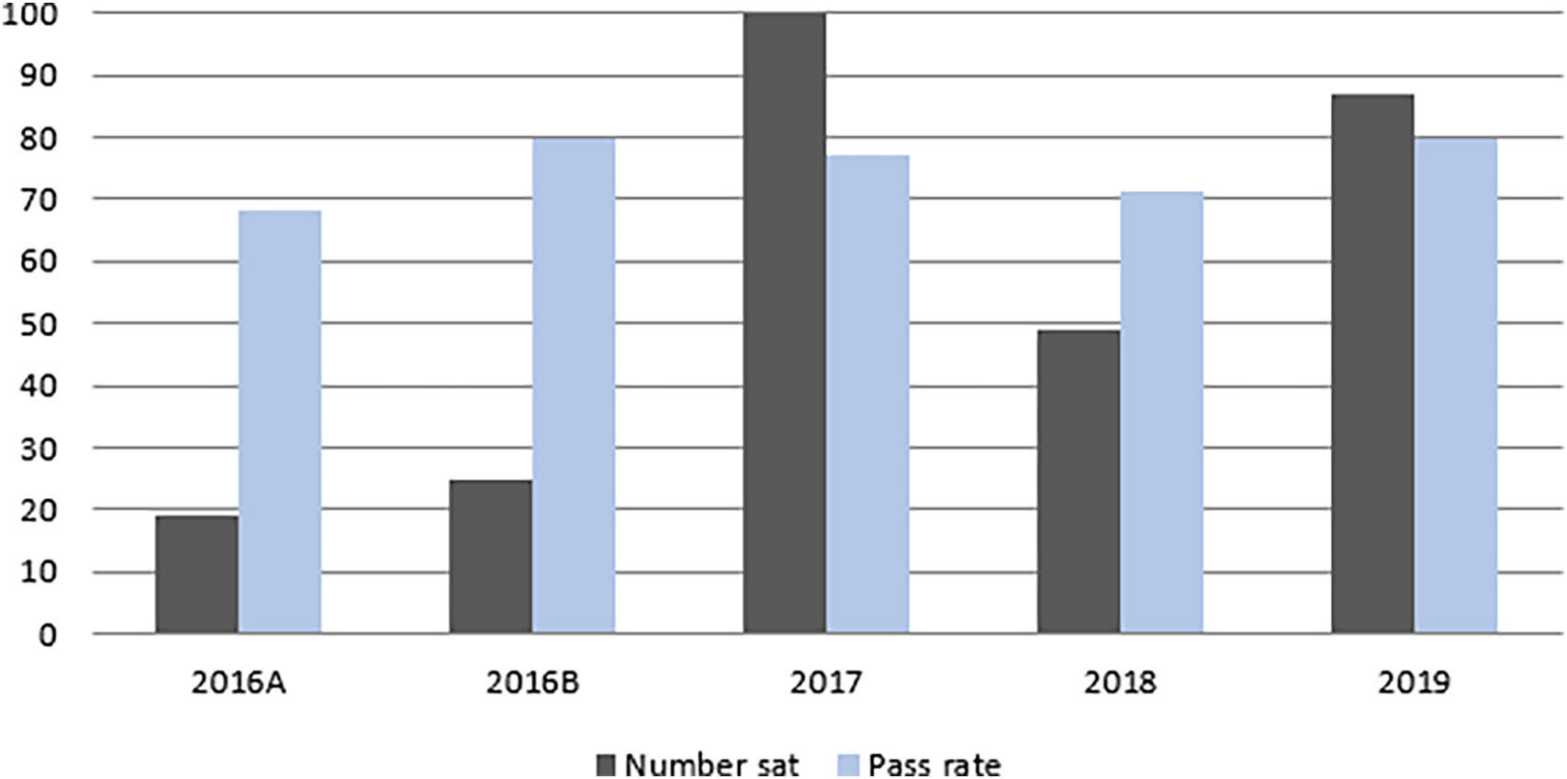
CBD pass rate and participation 2016- 2019

Figure 3 summarises CBD numbers and pass rates from 2016-2019. 280 candidates undertook the CBD over this period with an overall pass rate of 77%.

## Procedural Skills Logbook

The ACRRM Procedural Skills Logbook provides structured documentation of the candidate’s procedural experience. The Logbook contains those procedural items defined as ‘essential skills’ in the ACRRM Core Generalist Curriculum.

There are four different levels of competency described: assist; perform under supervision; perform in a simulated environment; perform independently. Some procedures can be completed and signed off at medical student level.

## Structured Assessments using Multiple Patient Scenarios (StAMPS)

StAMPS is a unique examination which blends the formats of an Objective Structured Clinical Examination (OSCE) and a traditional viva vocè examination. StAMPS scenarios are designed to reflect real life situations where clinical management must often proceed prior to a definitive diagnosis being made. They provide an opportunity for the candidate to explain the rationale behind their thinking ( Wilkinson *et al.*, 2008).

StAMPS is designed to test a candidate’s performance across the Core Generalist curriculum with consideration of the eight domains of rural and remote practice. It aims to assess that the candidate is demonstrating safe practice without supervision - not just demonstrating knowledge of the topic but considering the breadth and depth of the presentation and then contextualising management of the scenario into the rural/remote setting.

The format involveseight stations or scenarios delivered by eight examiners with each examiner delivering one scenario. Candidates receive written information about every scenario at the start of their 10-minute pre-exam reading time and can make notes. They have five minutes between scenarios to prepare for the next scenario.

Candidates have the option to undertake StAMPS via videoconference or face-to-face. In both formats candidates stay in one place (on a videoconference link or in one room) and the examiners rotate between rooms.

A Community Profile is developed each year to set the scene in which the candidates are expected to manage each scenario ( Australian College of Rural and Remote Medicine, 2020e). Candidates need to consider the location, demographics and the facilities available in the Community Profile in managing scenarios. Candidates need to consider the ‘constraints’ of the health service demographics and not answer questions from the perspective of their current working facility.

Examiners assess candidates in the following categories:

• Overall Impression
• Develop appropriate management plan that incorporates relevant medical and rural contextual factors
• Define problem systematically
• Communication
• Be flexible in response to new information

Grading is determined by using a matrix which evaluates the number of borderline and satisfactory marks in each category.

**Figure 4.**
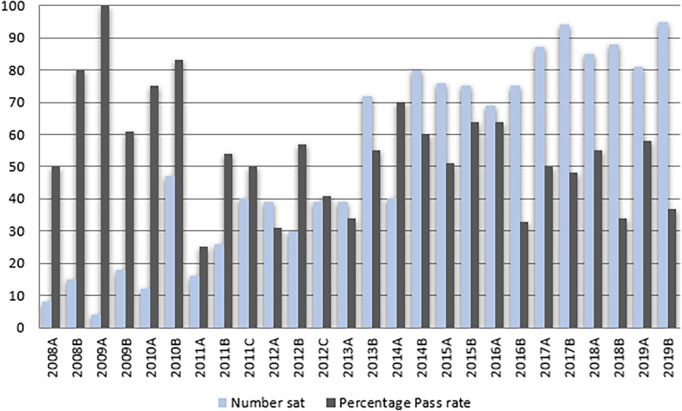
StAMPS pass rate and participation 2008 - 2019

Figure 4 summarises StAMPS candidate numbers and pass rates from 2008-2018. 1086 candidates undertook StAMPS over this period with an overall pass rate of 56%. Sample StAMPS Scenarios appear in Table 3.

**Table 3.** Sample Structured Assessment using Multiple Patient Scenarios (StAMPS) Scenario and Questions

**Scenario Information provided to Candidate:** Maria is a 59-year-old widowed cleaner who lives on her own in town. Her four children and five young grandchildren all live in the regional city 500km away. She was diagnosed with type 2 diabetes 10 years ago and hypertension five years ago. Her diabetes has been well controlled with her HbA1c usually in the range 7.0-7.5% (53-58mmol/mol) until the death of her husband thirteen months ago. Her blood pressure recordings over the past 2 years have all been <130/75 mmHg. She usually presents for regular review every three months after her routine pathology tests. Unusually, she failed to present for her most recent review and was recalled last week to have her overdue routine bloods done. Her usual medications include: Insulin glargine 30 units SC mane Metformin extended release 2g PO mane Gliclazide modified release 90mg PO mane Perindopril 5mg PO mane Aspirin 100mg PO mane Today, fingerprick random blood glucose is 22.0mmol/L, seated blood pressure is 165/90mmHg, From the pathology test taken last week her HbA1c was 10.8% (95mmol/mol) and her albumin: creatinine ratio was 28mg/mmol (normal range <3.5mg/mmol). Her weight has increased and her body mass index is now 37, up from 34 at her last visit five months ago. She tells you she stopped taking her insulin glargine a few weeks ago because it was making her feel puffy and sluggish. She avoids eye contact and appears untidy and exhausted. **This scenario has 3 Parts: estimated time for each Part: Part 1 - 1 minute, Part 2 - 6 minutes, Part 3 - 3 minutes** **Question 1**: Very briefly, what are the areas of concern that need to be addressed during this consult? **Question 2:** You’ve identified that depression may be a significant factor affecting Maria’s capacity for self care. How would you explore this further with her today? **Question 3:** What are the key issues regarding her diabetic control you would want to address?

## Exam Preparation and Information

A webpage for each assessment modality provides an overview of the assessment and sample questions supported by the Handbook for Fellowship Assessment ( Australian College of Rural and Remote Medicine, 2019). The ACRRM Registrar Committee has developed guides for candidates which outline useful information on ACRRM assessment for registrars by registrars.

The Assessment Program is blueprinted against the Core Generalist Curriculum ( Australian College of Rural and Remote Medicine, 2020c) with each assessment modality mapped to the relevant curriculum.

A report is published following each StAMPS and MCQ assessment. CBD public reports are issued each six months. The public report provides assessment statistics, a description of the scenarios/questions and feedback from the Lead Assessor, a summary of stakeholder feedback and planned improvements.

StAMPS study groups are held for the Core Generalist Curriculum and AST Emergency Medicine assessments. These study groups are held via online virtual classrooms, facilitated by experienced Medical Educators and are run in the weeks leading up to the assessment.

ACRRM offers mock assessments to allow candidates to practise StAMPS assessment questions under assessment conditions. Candidates are provided with feedback both face-to-face, and in writing. Accredited Training Organisations also provides assessment preparation programs.

## Organization and Governance

ACRRM’s assessments are overseen by a tight governance and management structure to ensure the process is fair, robust and transparent. This governance also ensures that decision-making processes and improvements are appropriately considered and approved. The Censor-in-Chief chairs the Board of Examiners. Responsibility for content and assessment processes rests with the Assessment Committee and the International Medical Graduate Committee, supported by numerous ACRRM staff including the General Manager Education Services and Assessment Manager. Lead Assessors for each modality oversee quality assurance processes and the conduct of each assessment. The ACRRM assessment team manages assessment administration.

ACRRM employs multiple quality assurance processes. There is a formal assessment blueprinting process with specifications outlining the number of questions that will be covered across each curriculum domain in each assessment. Assessment guidelines have been developed for case writers, staff and candidates.

Case writers are experienced rural doctors who hold the FACRRM. Writers are trained and supervised by the lead assessor who convenes a review and editing panel. Questions undergo significant road-testing and statistical analysis and are retired when necessary. Assessors must complete a substantial training and induction process

Other roles of the lead assessor include: assessment development; approval of assessor teams; conduct of training and assessor briefings and assessor moderation sessions; monitoring of assessment conduct and double marking; review of recordings and marking; review of special considerations and reconsiderations; post-examination statistical review; quality improvement processes; and evaluation of feedback

ACRRM employs a cohort of medical educators under the guidance of the Director of Training to specifically plan, monitor and facilitate training for candidates on the Independent Pathway. ACRRM’s medical educators are employed based on their educational and clinical expertise to ensure that educators with experience in a range of disciplines are available to support candidate training, assessment preparation and successful program completion.

## Lessons Learned

ACRRM’s Fellowship requirements, summarised in Table 1, represent a customised bespoke assessment tailored to ACRRM’s curriculum and the Australian rural and remote context. The assessment takes a programmatic approach with use of multiple modalities and feedback to candidates eg, through formative mini-CEXs and the MSF process. The assessments are designed to be undertaken by candidates in or near their own community.

**Figure 5.**
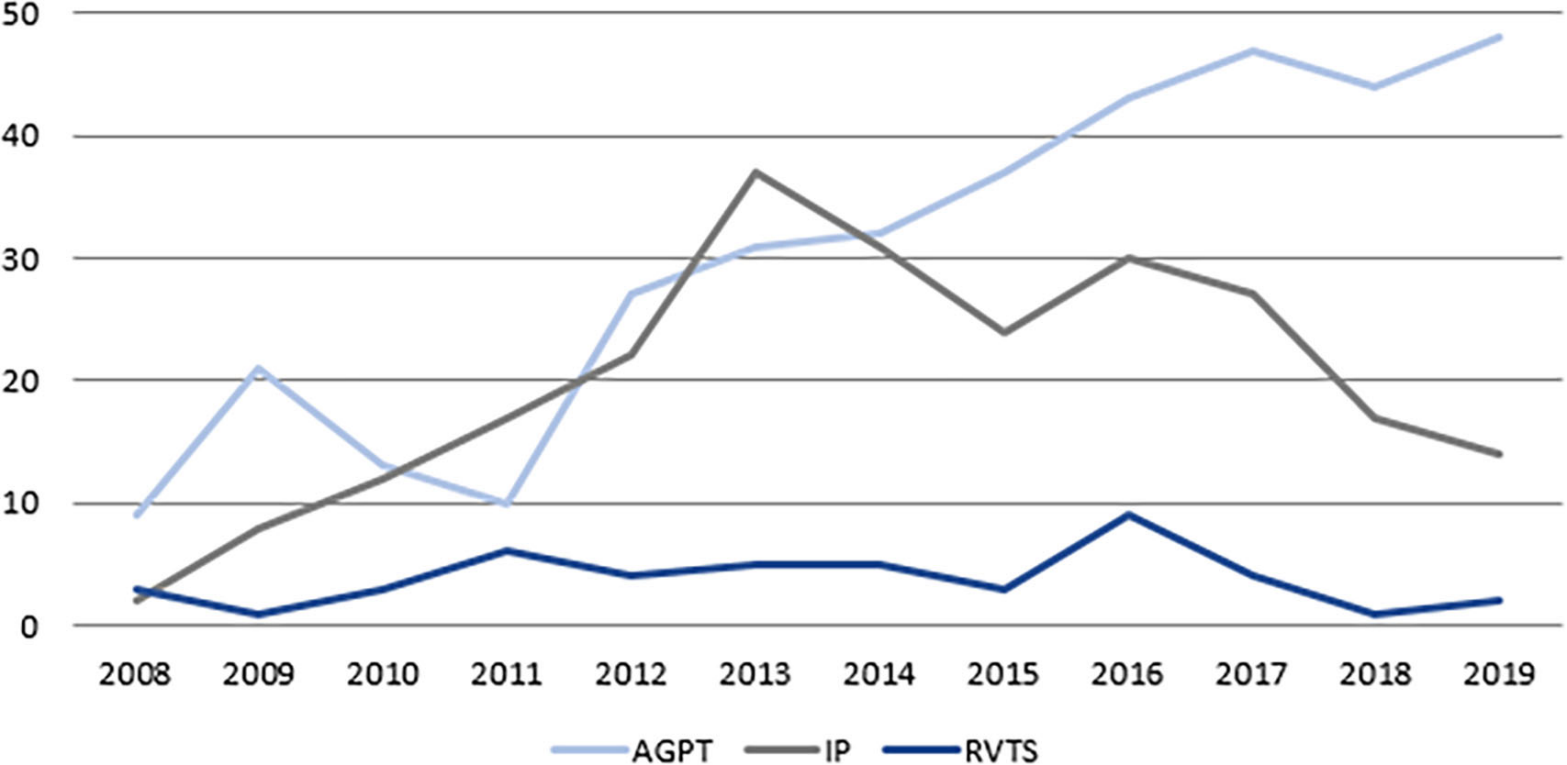
Fellowships awarded by year and cohort

**Figure 6.**
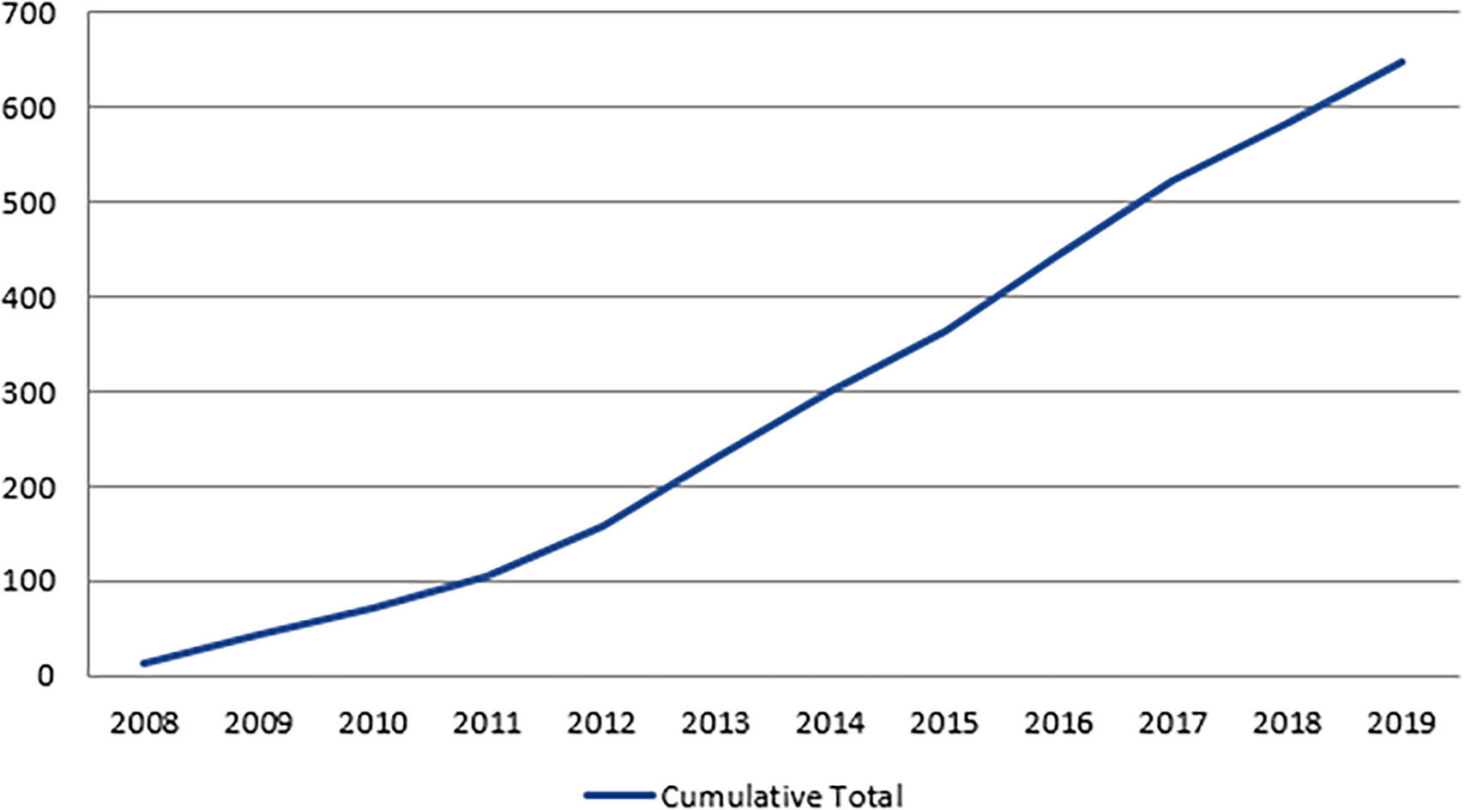
Cumulative total ACRRM Fellowship awards

Figures 5 and 6 demonstrate the growth in ACRRM’s assessment program, with 649 Fellowships being awarded between 2008 and 2019. The majority (56%) of these were via the Australian General Practice Training program, with 37% via the Independent Pathway, and 7% via the Remote Vocational Training Scheme.

Considerable experience in rural and remote assessment has been gained over the years, with expertise developed amongst staff and examiners. Many of the original candidates are now examiners or involved in developing and administering the assessment. Oversight by ACRRM Assessment committee has ensured the Fellowship exam has evolved over time in response to feedback and experience. With over 600 Fellows it now represents a mature firmly-established process as the world’s first Fellowship in Rural & Remote Medicine.

Significantly, ACRRM has continued to be able to offer its assessment program in 2020 despite the impact of the COVID-19 pandemic, perhaps uniquely among Australian medical colleges. The ‘tele-assessment’ design, allowing candidates and examiners to participate in assessment by use of distance technology while remaining in or near their home community may provide some insights for other medical Colleges and educational institutions facing challenges in the current environment.

## Take Home Messages

• The ACRRM Fellowship is the world’s first Fellowship exam in Rural Medicine.
• The assessment program is mapped to ACRRM’s Rural Generalist curriculum.
• Assessments are designed to be undertaken by candidates in or near their own community.
• Considerable experience has been gained in rural and remote assessment with 649 Fellowships being awarded from 2008 - 2019.
• The ‘tele-assessment’ model may provide some insights for other medical Colleges and educational institutions facing challenges in the current COVID-19 pandemic.

## Notes On Contributors

**Tarun Sen Gupta**: Chair, Assessment Committee, The Australian College of Rural and Remote Medicine. ORCID: https://orcid.org/0000-0001-7698-1413

**David Campbell**: Censor-in-Chief, The Australian College of Rural and Remote Medicine.

**Alan Bruce Chater**: Member, Assessment Committee, The Australian College of Rural and Remote Medicine.

**David Rosenthal**: Member, Assessment Committee, The Australian College of Rural and Remote Medicine.

**Lynn Saul**: Standards and Accreditation Manager, The Australian College of Rural and Remote Medicine.

**Karen Connaughton**: Assessment Manager, The Australian College of Rural and Remote Medicine, 2017-2020.

**Marita Cowie**: Chief Executive Officer, The Australian College of Rural and Remote Medicine.
