# Supplementary material for: Fellowship of the Australian College of Rural & Remote Medicine (FACRRM) Assessment: a review of the first 12 years
Source: MedEdPublish (2016). 2020 May 18;9:100. [Version 1] doi: 10.15694/mep.2020.000100.1 (PMC10712634; doi:10.15694/mep.2020.000100.1)
Supplement: Peer review history

# Reviewer report: Reviewer response for version 1

**DOI:** 10.21956/mep.20066.r30813

**Published:** 2020-05-21

**Authors:** Yingzi Huang

**Affiliations:** 1 The First Affiliated Hospital, Sun Yat-sen University

This review has been migrated. The reviewer awarded 4 stars out of 5 I enjoyed reading this informative article. I would though like to see more elements of formative assessment and how it's combined into the full picture of the programmatic assessment. And if the WBA pass rates are higher than for the more traditional methods, could it be due to the reason that people tend to pass candidates rather than fail them in work environment? If it's the case, then should entrustment and supervision scale be introduced to fit into the program, to ensure step-wise autonomy based on not just ability but also reliability, integrity and humility, which are all indispensible components leading to trust in candidates' competence as whole.

Reviewer Expertise:

NA

No decision status is available

# Reviewer report: Reviewer response for version 1

**DOI:** 10.21956/mep.20066.r30814

**Published:** 2020-05-20

**Authors:** Richard Hays

**Affiliations:** 1 James Cook University

This review has been migrated. The reviewer awarded 4 stars out of 5 This is a welcome paper, as it describes in more detail how an assessment system has performed and evolved over the few years since it was introduced. At the time it was regarded as pioneering and possibly 'risky', but experience has shown that the use of a broader range of assessment methods, many in the workplace, does contribute to high stakes decisions. I should disclose that I was involved a little in the early days in the development of an item bank for MCQs and in the design of the StAMPS clinical examination, and then after 10 years working in other fields have been asked to comment on quality assurance processes. This paper fills in many gaps in my understanding and lays down a more formal track record that many specialty training groups would avoid publishing. The only potential weakness I can see is that an assessment system that manages 600 successful candidates (plus an unknown number of unsuccessful candidates) over 12 years is not a huge logistic exercise. What plans are there to scale up or what advice could be provided for others considering implementation of a similar system on a larger scale? I have one further question - is it possible to calculate an overall success rate? The pass rates for individual components are said to be 68, 71, 91 and 56%, but I suspect that the denominator may be hard to calculate due to the different pathways to assessment. Related to that, the WBA pass rates are higher than for the more traditional methods - this is often seen in WBA. What implications might that have for eligibility, sequencing and combining of individual methods? Dealing with these issues would merit an extra star. The message may be contextualised in Australia, but there may be implications for training programs elsewhere.

Reviewer Expertise:

NA

No decision status is available

# Reviewer report: Reviewer response for version 1

**DOI:** 10.21956/mep.20066.r30812

**Published:** 2020-05-19

**Authors:** Balakrishnan Nair

**Affiliations:** 1 Centre for Medical Professional Development and University of Newcastle

This review has been migrated. The reviewer awarded 5 stars out of 5 I enjoyed reading this paper on WBA. It is good to see a programmatic assessment in postgraduate education in remote medicine. The authors describe the evolution of this format over a period of 12 years, graduating 649 Fellows. They have used the standard tools of Workplace-based assessment ( mini cex, CBD, logbook and MSF) in addition to MCQs and structured viva. It will be interesting to study reasons for the low pass in Minicex and the why they did not assess record-keeping in CBD(which is traditionally done). The strength of the programmatic assessment is feedback after every assessment, and I would have preferred to see some discussion about this There are many lessons from this long-standing program.

Reviewer Expertise:

NA

No decision status is available

# Reviewer report: Reviewer response for version 1

**DOI:** 10.21956/mep.20066.r30815

**Published:** 2020-05-18

**Authors:** Felix Silwimba

**Affiliations:** 1 University of Lusaka

This review has been migrated. The reviewer awarded 5 stars out of 5 Well explained easy to follow article. relevant to my settings in a low resources country. I recommend for other medical educators in low income countries to read it.

Reviewer Expertise:

NA

No decision status is available
